# *CAPRIN1* Pro512Leu Variant Causes Childhood Dementia, Myoclonus‐Ataxia, and Sensorimotor Neuropathy

**DOI:** 10.1002/mdc3.14347

**Published:** 2025-01-29

**Authors:** Rossella Bove, Annalaura Torella, Maria Novelli, Giacomina Ricciardi, Luca Pollini, Laura Masuelli, Roberto Bei, Mariateresa Zanobio, Francesco Pisani, Vincenzo Nigro, Vincenzo Leuzzi, Serena Galosi

**Affiliations:** ^1^ Child Neurology and Psychiatry Unit, Department of Human Neurosciences Sapienza University of Rome Rome Italy; ^2^ Department of Precision Medicine University of Campania “Luigi Vanvitelli” Naples Italy; ^3^ Department of Experimental Medicine Sapienza University Rome Italy; ^4^ Department of Clinical Sciences and Translational Medicine University of Rome Tor Vergata Rome Italy

**Keywords:** cell cycle‐associated protein 1, ataxia, neuropathy, cognitive decline

The *CAPRIN1* gene (OMIM * 601178) encodes for the Cell Cycle‐Associated Protein 1 (*CAPRIN1*), a ubiquitously expressed protein that is highly enriched in tissues with elevated cell turnover^1^, ^2^, ^3^ and in the central nervous system (CNS), particularly in the frontal cortex and cerebellar hemispheres.^4^ In the CNS, *CAPRIN1* acts as an RNA‐binding protein and regulates the transport and translation of mRNAs of several synaptic proteins.^2^, ^5^

A total of 15 patients with two different early‐onset neurological phenotypes, a neurodevelopmental disorder on one side and a progressive neurodegenerative one on the other, have been reported so far.^6^, ^7^

Here, we contribute to the clinical characterization of this ultra‐rare disease with a new pediatric patient with a severe neurodegenerative phenotype.

This 13‐year‐old girl was born at term by caesarean section for premature rupture of membranes and oligohydramnios after an uneventful pregnancy. She is the only child of non‐consanguineous parents. Her mother is healthy, while her father died prematurely of acute myocardial infarction. Several cases of intellectual disability (ID) and unspecified neurological conditions have been reported in the paternal line of the family. Clinical history was uneventful until the age of 8 years, when she presented with insidious motor clumsiness, evolving into gait instability and recurrent falls by the age of 10. By this time, cognitive performance declined, with learning and language difficulties and a total intelligence quotient of 65 on a formal cognitive assessment. At her first examination at the age of 11, she presented with mild generalized ataxia, bradykinesia, osteotendinous hyporeflexia, and mild muscle atrophy (Video 1). Spontaneous and evoked arrhythmic muscle jerks were observed in the upper limbs and were unrelated to the bilateral spike–wave complexes recorded by electroencephalograms (Fig. S1). Two subsequent brain magnetic resonance imaging (MRI) scans over 6 months showed progressive frontal lobe and cerebellar atrophy (Fig. 1A,B). Electroneurography confirmed sensorimotor axonal neuropathy in the lower and upper limbs. Progression of ataxia and neuropathy led to loss of independent walking by the age of 13 years (Video 2). At this age, response to a levodopa trial (3 mg/kg) was observed, for about 5 months, concerning trunk instability, bradykinesia (Video 3), swallowing, and speech fluency.

**Video 1:** The video shows the patient at 10 years and 8 months when she first came to our attention. She was ataxic but still able to walk independently. She could stand with slight instability with closed eyes. She showed postural instability with feet in a tandem position (right > left). Running was clumsy, with dystonic postures of the upper limbs. Her speech was dysarthric. The finger‐to‐nose test revealed dysmetria (right > left). A fine tremor with jerky intrusions was observed in the upper limbs. Finger tapping was impaired by lack of coordination, fatigue, and bradykinesia.

**Figure 1:**
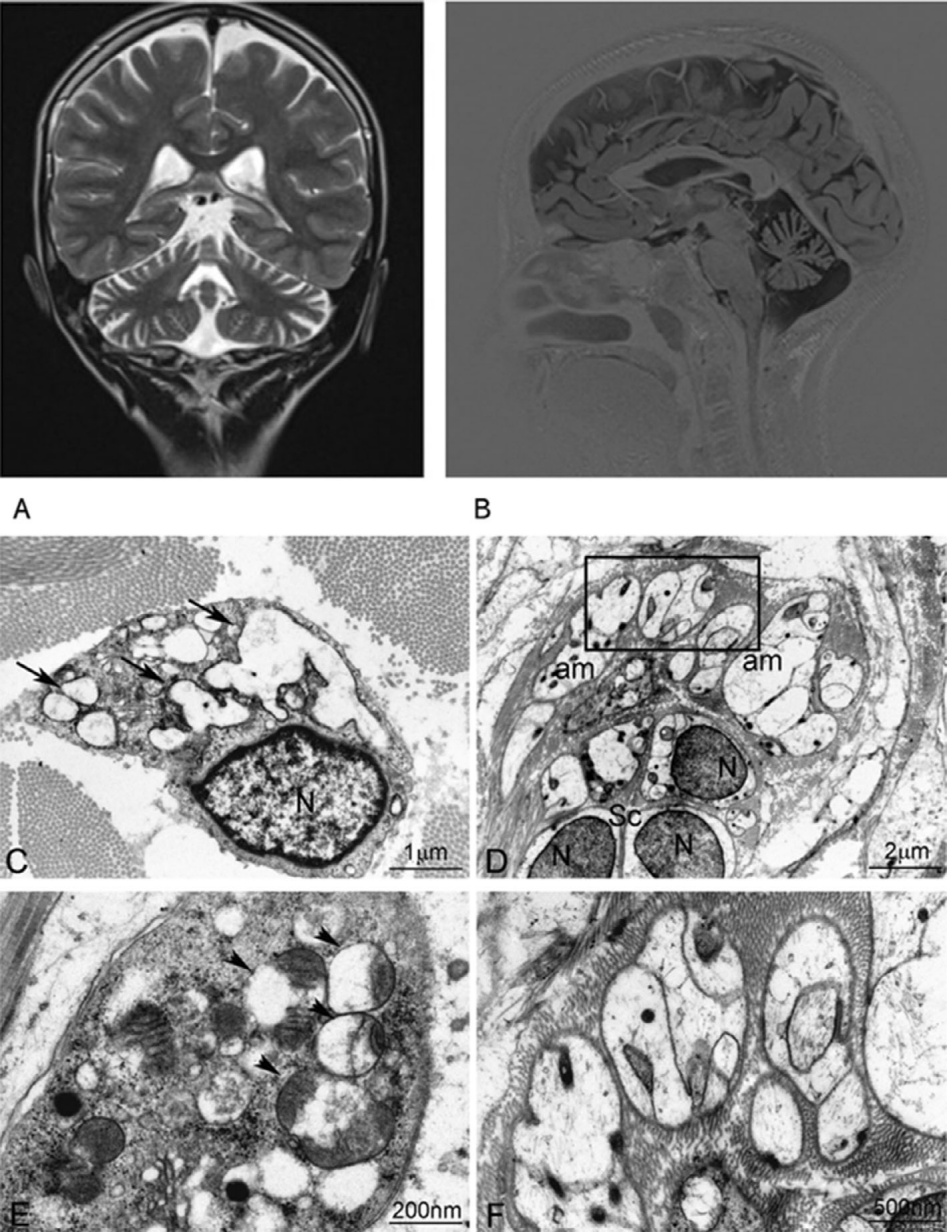
Brain MRI, coronal (A), sagittal (B); Ultrastructural analysis of skin biopsy. Swelling of the rough endoplasmic reticulum (arrows) in dermal fibroblasts (C), with mitochondrial alteration (arrowheads) (E); amyelinated nervous fibers show axon swelling at low (D) and higher magnification (F). N, nucleus; am, amyelinated nervous fibers; Sc, Schwann cells.

**Video 2:** Age: 12 and 13 years. Rapid worsening of ataxia, hypotonia, brady‐ and hypokinesia. Postural and action tremors show a higher amplitude with myoclonic jerk intrusions. The girl became wheelchair‐bound.

**Video 3:** The patient before (Segment 1) and after treatment with levodopa (Segment 2). She experienced mild improvement in bradykinesia.

Ultrastructural analysis of the skin biopsy showed swelling of the rough endoplasmic reticulum (RER), mitochondrial changes in dermal fibroblasts (Fig. 1C,E), and swelling of unmyelinated nervous fibers (Fig. 1D,F). Whole exome sequencing identified a previously reported c.1535C > T (p.Pro512Leu) missense variant in *CAPRIN1*.^6^ The patient's mother did not carry the variant. Other relatives were either unavailable (father) or did not consent to genetic examination.

*CAPRIN1* haploinsufficiency has been identified as a cause of neurodevelopmental disorders since 2017.^7^, ^8^, ^9^ Twelve patients have been described: language impairment was observed in all cases, variably associated with ID, autistic features, and attention deficit hyperactivity disorder (ADHD); four patients had epilepsy, two with absence seizures, one with infantile spasms at 9 months of age followed by absences, and one with focal seizures with secondary generalization.^7^ All patients achieved discrete seizure control with pharmacological treatment but developed ID.^7^

More recently, a severe neurodegenerative presentation associated with the Pro512Leu gain‐of‐function variant was reported in two index patients who presented at the ages of 10 and 7 years with progressive ataxia, cognitive decline, sensorimotor axonal neuropathy, and cerebral and cerebellar atrophy.^6^ One more patient with the same variant and an identical phenotype was notified to the authors after publication.^6^ The functional study suggested that the Pro512Leu substitution renders *CAPRIN1* protein prone to misfolding and aggregation, resulting in the sequestration and inactivation of several cognate proteins, implicated in ataxic and neurodegenerative diseases, including *ATXN2*, *GEMIN5*, *SNRNP200*, and *SNCA*. Impairment of multiple gene products explains the severity of the phenotype. The inability of the protein quality control machinery to effectively manage mutant protein aggregation is believed to lead to chronic endoplasmic reticulum stress, which can disrupt cellular homeostasis and contribute to various pathological conditions.^6^ RER stress has been associated with the mitochondrial dysfunction observed in aging or pathological conditions.^10^ This is suggested in our case by the swelling of fibroblast rough endoplasmic reticulum. Moreover, we detected morphological alterations of the mitochondria in skin fibroblasts and ultrastructural changes in peripheral nerve fibers. The quick progression of motor impairment associated with dementia without any prior neurodevelopmental disorder may suggest a diagnosis overlapping with conditions resulting from isolated involvement of each of the proteins affected by *CAPRIN1* defect. In conclusion, the present case confirms the Pro512Leu variant on *CAPRIN1* as a genetic lesion causing a new specific early‐onset neurodegenerative disease presenting with progressive ataxia and dementia.

## Author Roles

(1) Research project: A. Conception, B. Organization, C. Execution; (2) Statistical Analysis: A. Design, B. Execution, C. Review and Critique; (3) Manuscript Preparation: A. Writing of the First Draft, B. Review and Critique.

R.B.: 1B, 1C, 3A, 3B.

A.T.: 1C, 3A, 3B.

M.N.: 1C, 3B.

G.R.: 1C, 3B.

L.P.: 1C, 3B.

L.M.: 1C, 3B.

R.B.: 1C, 3B.

M.Z.: 1C, 3B.

F.P.: 1C, 3B.

V.N.: 1C, 3B.

V.L.: 1A, 1B, 1C, 3A, 3B.

S.G.: 1A, 1B, 1C, 3A, 3B.

## Disclosures

**Ethical Compliance Statement:** The authors confirm that the approval of an institutional review board was not required for this work. Written informed consent for offline and online distribution of the video material was obtained from parents and is available on request. We confirm that we have read the Journal's position on issues involved in ethical publication and affirm that this work is consistent with those guidelines.

**Funding Sources and Conflicts of Interest:** No specific funding was received for this work. The authors declare that there are no conflicts of interest relevant to this work.

**Financial Disclosures for Previous 12 Months:** The authors declare that there are no additional disclosures to report.

## Supporting information


**Figure S1.** Awake EEG showing subclinical anterior bilateral spike–wave complexes.

## Data Availability

The data that support the findings of this study are available on request from the corresponding author. The data are not publicly available due to privacy or ethical restrictions.
