# Supplementary figures and images for: CAPRIN1 Pro512Leu Variant Causes Childhood Dementia, Myoclonus‐Ataxia, and Sensorimotor Neuropathy
Source: Mov Disord Clin Pract. 2025 Jan 29;12(5):694–8. doi: 10.1002/mdc3.14347 (PMC12070162; doi:10.1002/mdc3.14347)

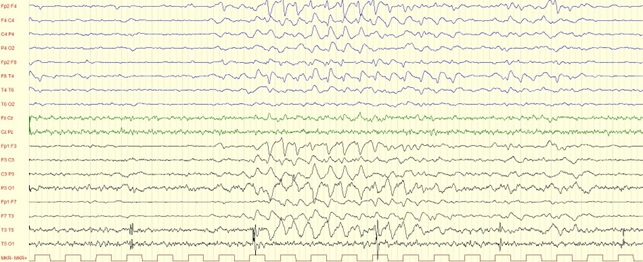

Supplement: Supplementary file 1 — Figure S1. Awake EEG showing subclinical anterior bilateral spike–wave complexes. [file MDC3-12-694-s001.png]
